# Near-Infrared Light-Responsive Molybdenum Disulfide Nanosheets for Controlling the Release of Nimodipine as NIR-Drug Delivery System

**DOI:** 10.3390/molecules30030497

**Published:** 2025-01-23

**Authors:** Mohamed M. Abdelghafour, Ágota Deák, Keristina Wagdi K. Amin, Zsófia Czimer, Czike Flóra Veronika, Viktória Péter, Róbert Berkecz, Ferenc Bari, László Janovák

**Affiliations:** 1 Department of Chemistry, Faculty of Science, Zagazig University, Zagazig 44519, Egypt; m.abdelghafour2015@yahoo.com; 2 Department of Physical Chemistry and Materials Science, University of Szeged, Rerrich Béla tér 1, H-6720 Szeged, Hungary; agotadeak@chem.u-szeged.hu (Á.D.); zsofia010105@gmail.com (Z.C.); 3 Department of Chemistry, Faculty of Science, Suez Canal University, Ismailia 41522, Egypt; 4 Department of Medical Physics and Informatics, Faculty of Medicine and Faculty of Science and Informatics, University of Szeged, Korányi ´ Fasor 9, H-6720 Szeged, Hungary; czfloravera@gmail.com (C.F.V.); peter.viktoria@med.u-szeged.hu (V.P.); bari.ferenc@med.u-szeged.hu (F.B.); 5 Institute of Pharmaceutical Analysis, Faculty of Pharmacy, University of Szeged, Somogyi utca 4, H-6720 Szeged, Hungary; berkecz.robert@szte.hu; 6 Department of Forensic Medicine, Albert Szent-Györgyi Health Center, Kossuth Lajos sgt. 38, H-6724 Szeged, Hungary

**Keywords:** chitosan, nimodipine, near-infrared (NIR) light responsive, drug release control

## Abstract

Here, we present a photothermally triggered drug delivery nanosystem MoS_2_-NIMO-CHIT-SH, using the thiolated chitosan (CHIT-SH)-modified molybdenum disulfide (MoS_2_) nanosheets as near-infrared (NIR) photo-responsive carriers, loaded with the dihydropyridine calcium antagonist drug Nimodipine (NIMO). Although NIMO is used to treat stroke, migraine, Alzheimer’s disease, cerebrovascular spasms, and hypertension, this drug is poorly water-soluble, with low bioavailability and lack of selectivity. Thus, there is an urgent need for a novel approach to creating NIMO formulations that are safe, effective, and have better solubility and bioavailability. To overcome these problems, we develop a cationic biopolymer functionalized MoS_2_ nanosheets as a photothermal drug carrier system to facilitate the NIR light-induced release of NIMO drugs. MoS_2_ nanosheets (<150 nm) as NIMO drug carriers are prepared through simple exfoliation of their bulk phase and then functionalized with CHIT-SH biopolymer to increase their physiological stability and biocompatibility. According to the results, MoS_2_-NIMO-CHIT-SH nanocomposites show strong NIR absorbance, which makes them a promising candidate for photothermal therapy.

## 1. Introduction

One of the most prevalent polysaccharides in nature, chitin, is alkalinely deacetylated to produce chitosan (CHIT). Glucosamine and N-acetyl glucosamine combine to form chitosan (CHIT), a naturally occurring cationic polymer with advantageous properties, including mucoadhesion, biocompatibility, biodegradability, antimicrobial activity, and the capacity to open tight junctions [1]. CHIT is a cationic biopolymer with low toxicity, biocompatibility, and biodegradability, making it a suitable drug carrier choice. Additionally, CHIT has the ability to increase penetration and has garnered a lot of interest due to its potential to improve absorption for transport across mucosal epithelia. But at pH values higher than 6, CHIT loses its positive-charge density, becomes poorly soluble in water, and causes precipitates and aggregates to form, all of which reduce its efficiency at absorption sites. Thiolated chitosan (CHIT-SH), which is made up of a new class of biopolymers called thiolated polymers or thiolmers [2], was developed in order to address this issue. Furthermore, the various thiolate ligands were used for the exfoliation of MoS_2_ nanosheets, a well-known and widely used transition metal dichalcogenides (see later) since the exfoliated 2D-MoS_2_ contains both Mo and S defects [3]. Due to Mo defects, 2D-MoS_2_ will have dangling thiols, which help to form disulfide bonds with external thiols. Alternatively, S defects lead to the formation of metal–sulfur bonds. Therefore, in both cases, exfoliated 2D-MoS_2_ has a high affinity toward the external thiols. Hence, various thiolate ligands were used for functionalization. In this current study, we chose 3-mercaptopropionic acid as the thiolated reagent for the synthesis of CHIT-SH.

Stimulus-responsive polymers based on ultrasound [4,5,6], magnetic field [7,8], enzymes [9], electrical field [10], pH [11,12,13,14], temperature [15], and light [16,17,18,19,20,21] stimuli have been the subject of extensive research recently for the triggered release of hydrophobic drugs in controlled drug delivery systems. This is because of a number of benefits, including a significant increase in drug water solubility, a longer circulation time, a decrease in side effects, and an improvement in drug bioavailability [22]. Since the release process may be remotely activated and the drug release can be controlled spatially or temporally to occur at a chosen moment by illumination, photo-cleavage has garnered more attention than temperature- or pH-sensitive techniques. The majority of light-responsive photochromic moieties, however, react to UV or visible light, whose energy may be readily depleted by tissues in vivo and can seriously harm human health, limiting their potential usage in biological or biomedical applications [23,24].

Longer wavelengths of near-infrared (NIR) light, roughly 700–1000 nm, are less harmful to healthy cells and can penetrate tissue more deeply because they are less absorbed and scattered by biological materials and water [22]. Because it has less tissue absorption, more effective tissue penetration, less scattering, and less autofluorescence [25,26], near-infrared (NIR) light is frequently utilized in the treatment of diseases, including cancer photothermal therapy [27] and diagnostic imaging [26]. This is mostly because of its unique surface-area-to-mass ratio, higher absorption intensity in the near-infrared (NIR) region compared to graphene and gold nanorods, solubility in water after stripping, ease of purification of exfoliated MoS_2_ nanosheets, and high photothermal conversion efficiency in the NIR region [28]. Liu et al. employed the photothermal conversion capabilities of MoS_2_ nanosheets to provide chemotherapy and photo thermotherapy for cancer [29]. MoS_2_ nanosheets have been employed in additional investigations as a photothermal agent and drug carrier for antibacterial research [30,31], diagnostic imaging [32,33], and cancer treatment [27,28,32,33,34].

A dihydropyridine calcium antagonist, Nimodipine (NIMO), is used to treat stroke, migraine, Alzheimer’s disease, cerebrovascular spasm, and hypertension [35,36,37,38]. Although NIMO is now utilized in clinical settings through oral soft gelatin capsules and oral tablets, its therapeutic effectiveness is limited due to a number of negative aspects, including high dosages, poor oral bioavailability, and many side effects [39,40,41]. Additionally, NIMO is a weakly water-soluble medication that readily breaks down in the presence of light, losing its pharmacological efficacy [35,42]. Thus, there is an urgent need for a novel approach to creating NIMO formulations that are safe, effective, and have better solubility and bioavailability.

In this paper, a drug delivery nanosystem MoS_2_-NIMO-CHIT-SH was constructed using the thiolated chitosan (CHIT-SH) and MoS_2_ nanosheets as NIR photo-responsive carriers and loaded with the calcium channel blocker drug Nimodipine (NIMO). Loading NIMO on the MoS_2_-CHIT-SH nanoparticles can control the release by using NIR laser light.

## 2. Results and Discussion

**Figure 1:**
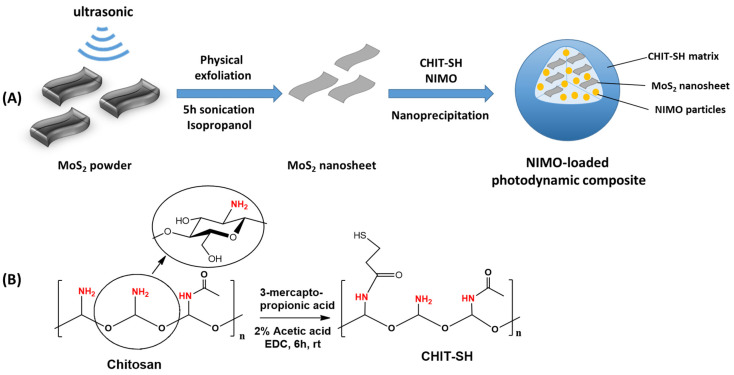
(**A**) Synthesis process of the NIMO-loaded photodynamic composite; (**B**) scheme of modification of chitosan with 3-mercaptopropionic acid to produce thiolated chitosan.

The objective of this work is to develop suitable NIR photo-responsive drug carriers for NIMO (Figure 1A) to decrease its adverse effects and improve its bioavailability. Despite having excellent levels of biocompatibility and biodegradability [43,44], CHIT’s poor solubility at physiological pH makes it unsuitable for use in the production of drug delivery systems. To increase the water solubility of CHIT, it was conjugated with 3-mercapto propionic acid to produce CHIT-SH (Figure 1B). Furthermore, the thiol moiety can also increase the exfoliation process of MoS_2_ nanosheet [3], which we employed as a carrier to initiate drug release based on the NIR photothermal conversion property of MoS_2_. Based on the nanoprecipitation strategy (Figure 1A), NIMO and MoS_2_ nanosheets were encapsulated within biocompatible and biodegradable CHIT-SH to produce NIMO-loaded photodynamic composite (NIMO-MoS_2_-CHIT-SH). This can improve the bioavailability of poorly water-soluble NIMO by reducing the drug’s crystallinity, which results in a higher dissolution rate and increased stability in an aqueous medium. Furthermore, the release of NIMO from the photodynamic composite can be modulated by the application of NIR light.

**Figure 2:**
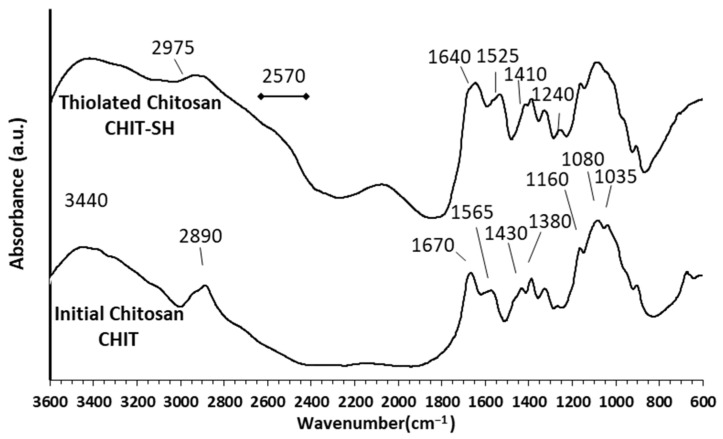
FTIR spectra of initial chitosan (CHIT) and thiolated chitosan (CHIT-SH).

The successful modification of initial chitosan (CHIT) to produce thiolated chitosan (CHIT-SH) was confirmed by FTIR measurements, as shown in Figure 2. Initial chitosan (CHIT) was demonstrated to have main distinctive peaks at 3440 and 2890 cm^−1^ are attributed to the stretching vibrations of –OH and –NH_2_ group, C–H stretching vibration, respectively, while the detected peaks at 1670 and 1565 cm^−1^ related to the C=O stretching of the amide I band, the bending vibrations of the N–H (N-acetylated residues, amide II band), respectively [45,46,47]. Moreover, the absorbance peaks at 1430, 1380, 1160, 1080, and 1035 cm^−1^ are attributed to the C–H bending, the O–H bending, the anti-symmetric stretching of the (C–O–C) bridge, and the skeletal vibration involving the C–O stretching are all attributed to these peaks, respectively [45,46,47]. The decrease in the intensity of the peak at 3440 cm^−1^, which is attributed to the –OH and –NH_2_ groups, the appearance of a new C–H stretching vibration peak of the 3-mercaptopropionate group at 2975 cm^−1^, and the broad peak at 2570 cm^−1^, which is related to the S–H group, confirmed the success of the substitution reaction and grafting of 3-mercaptopropionic acid onto chitosan. C-SH stretching is the cause of the new peak at 1240 cm^−1^ [48,49]. The conjugation of 3-mercaptopropionic acid to NH_2_ of chitosan results in a shifting of the amide I band peak, which decreases by approximately 30 cm^−1^, as well as the emergence of a new peak near the amide II band at 1525 cm^−1^ [48,49].

**Figure 3:**
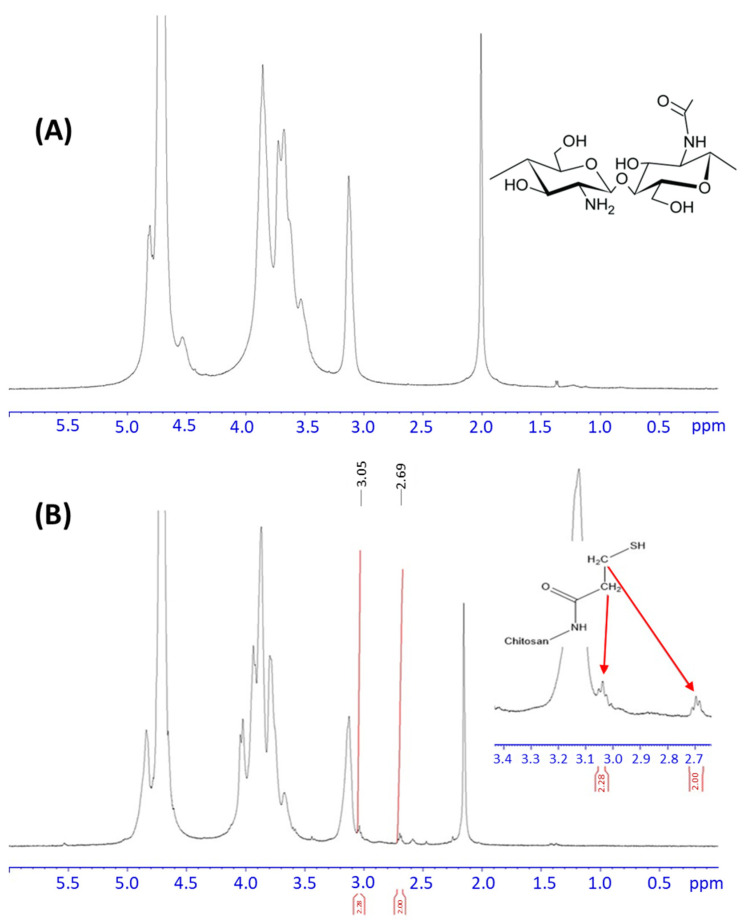
^1^H-NMR spectra of (**A**) Chitosan (upper); (**B**) thiolated chitosan (CHIT-SH; lower).

The ^1^H-NMR spectra were also recorded (see Figure 3) following the FTIR chemical characterization of CHIT and CHIT-SH. The CHIT-SH spectrum displays the typical chitosan peaks (Figure 3A) as well as two additional peaks at 2.69 and 3.05 ppm that we believe are related to the 3-mercaptopropionate moiety (Figure 3B).

^1^H-NMR evaluations showed that the degree of substitution (DS%) of chitosan with 3-mercaptopropionic acid was 4.43%, while the degree of deacetylation (DD) of the original chitosan was equivalent to 75.02% [43]. Based on the collected polymers and the CHIT-SH product’s degree of substitution, the typical yield was approximately 84% [43].

**Figure 4:**
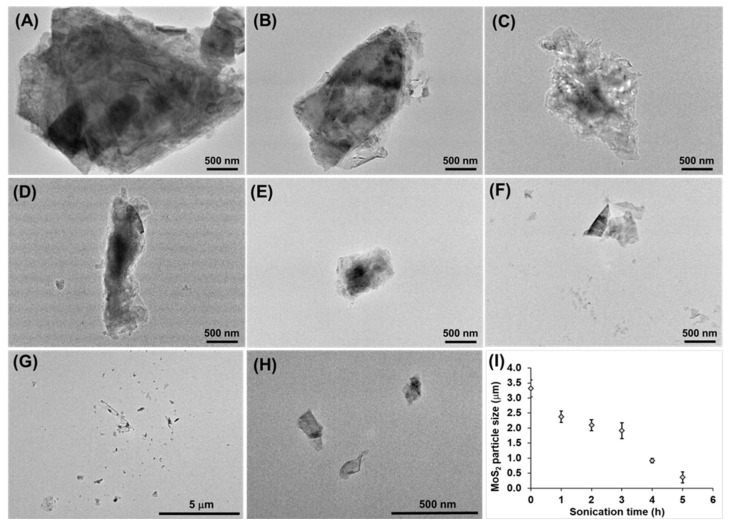
(**A**) TEM micrographs of the initial MoS_2_ (without sonication) and after (**B**) 1, (**C**) 2, (**D**) 3, (**E**) 4, and (**F**) 5 h of sonication time. TEM micrographs of the MoS_2_ nanosheets (**G**) before and (**H**) after 10 min of centrifugation. (**I**) The average particle size of the MoS_2_ sheets as a function of sonication time.

To obtain MoS_2_ nanosheets, a liquid phase exfoliation technique was used. Figure 4 presents the TEM micrographs of the initial (powder form) MoS_2_ (without sonication) and after 1, 2, 3, 4, and 5 h of sonication time. The initial MoS_2_ size decreases gradually from ~3.32 mm after ultrasonic treatment. After 1 and 2 h of sonication, it can be observed that the size decreases to 2.37 ± 0.19 µm and 2.09 ± 0.18 µm. Upon further treatment, after 3 h of sonication, a further size reduction is observed (1.91 ± 0.26 µm). The greatest size reduction was achieved after 4 and 5 h of ultrasonic treatment, during which we obtained particles with sizes of 917 ± 78.7 nm and 359 ± 18.2 nm. Our results were consistent with previous published work [50].

A low-speed centrifugation was applied to separate the exfoliated MoS_2_ nanosheets from the larger particles (Figure 4G). After 10 min of centrifugation (1500 rpm), 140 ± 19.2 nm MoS_2_ nanosheets were obtained (Figure 4H).

Given the growing prevalence of these 2D materials in daily life and their increasing use in a variety of practical applications, it is crucial to look into the toxicity, environmental impact, and biocompatibility of MoS_2_ onto various human cell lines. In order to initiate and maybe regulate the surface contacts of MoS_2_ nanosheets onto living matter, some crucial criteria include the morphology, size, and thickness of 2D nanosheets. To harness the potential of 2D nanosheets in a range of biomedical applications, including drug delivery, cancer diagnosis, and cell imaging, functionalized MoS_2_ nanosheets have been investigated using various human cell lines [51,52,53]. For example, Siepi and coworkers reported on the biocompatible lysozyme-functionalized exfoliated MoS_2_ nanosheets, and they cultured MoS_2_ nanosheets on two distinct cell lines (HaCaT and HeLa), wherein at greater doses of the MoS_2_ nanosheets that showed no signs of harm [54]. Coleman and colleagues reported on the size and concentration-dependent toxicity of MoS_2_ nanosheets on three different established cell lines [55]. Appel and coworkers reported on the interactions of bare MoS_2_ nanosheets, obtained by mechanical exfoliation and chemical vapor deposition, with human epithelial kidney cells (HEK293f) and found that there was little cytotoxicity and genotoxicity in their experimental conditions [56]. Shah et al. investigated the effect of MoS2 nanosheets produced by liquid exfoliation on rat cells, and they found a relatively good biocompatibility at high 2D material concentration [57].

**Figure 5:**
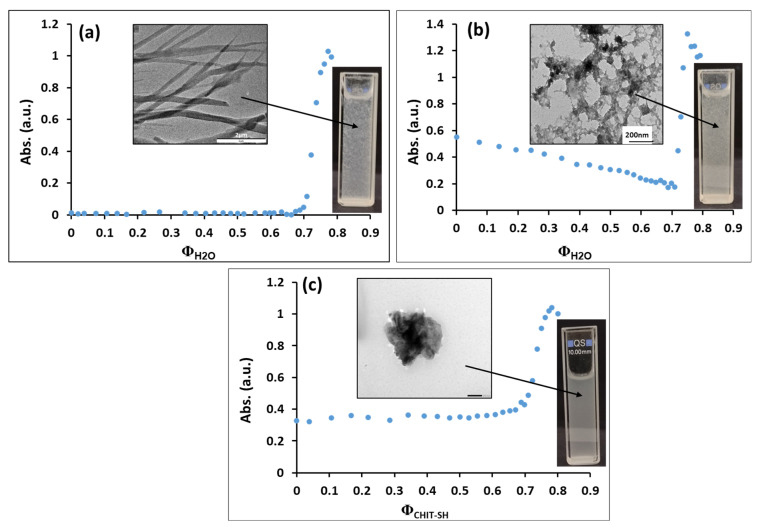
(**a**) The precipitation of ethanol-based NIMO solution (c = 0.1%) by distilled water; (**b**) the precipitation of ethanol-based NIMO (c = 0.1%) in the presence of MoS_2_ nanosheets (c = 0.00145%) by distilled water; (**c**) the precipitation of ethanol-based NIMO (c = 0.1%) in the presence of MoS_2_ nanosheets (c = 0.00145%) by aqueous CHIT-SH solution (c = 0.5%).

The solubility/precipitation properties of the NIMO and CHIT-SH were studied in the EtOH/H_2_O system to find a good strategy for producing a NIMO-loaded photodynamic composite by a simple precipitation method, as we previously reported [58]. NIMO (as a poorly water-soluble drug) shows good solubility in EtOH and can be precipitated by H_2_O, as presented in Figure 5a. The precipitation of ethanol-based NIMO solution required a large amount of water (~Ф_H2O_ = 0.68) to begin precipitation of micron-sized needle-like crystals of bare NIMO, even in the presence of MoS_2_, the precipitation of NIMO and MoS_2_ composite was occurring at (~Ф_H2O_ = 0.7) to form microstructure of composite as shown in Figure 5b.

However, as seen in Figure 5c, the precipitation of NIMO and MoS_2_ using 0.5% *w/v* of aqueous CHIT-SH solution showing the precipitation at (~Ф_CHIT-SH_ = 0.7) to generate NIMO-loaded photodynamic composite with spherical shape and particle size at around 500 nm.

**Figure 6:**
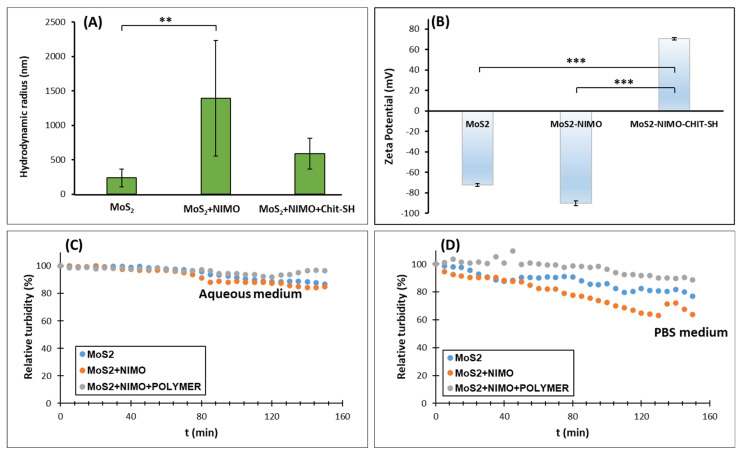
(**A**) Hydrodynamic radius for MoS_2_ nanosheet, MoS_2_-NIMO, and MoS_2_-NIMO-CHIT-SH composite; (**B**) the corresponding zeta-potential values of the samples. The time-dependent turbidity values of MoS_2_ nanosheet, MoS_2_-NIMO, and MoS_2_-NIMO-CHIT-SH composite dispersion (0.01% *w*/*v*) in (**C**) water and in (**D**) PBS medium. The particle size and the zeta potential measurements were statistically analyzed using a one-way ANOVA test followed by Tukey’s HSD post hoc test. The following designation was used for the levels of significance: ** *p* < 0.01; *** *p* < 0.001.

Particle size and stability of the formed particles were studied using DLS and turbidimeter measurements, as shown in Figure 6. The hydrodynamic radius of the MoS_2_ nanosheet was 237 ± 128 nm compared to the NIMO-MoS_2_ composite, which has 1392 ± 841 nm (Figure 6A). The reason for the significant increase in the particle size is due to precipitation of NIMO in microscale size. However, the presence of CHIT-SH biopolymer in the precipitation process led to a significant reduction in the standard deviation of the particle size (588 ± 221 nm), as shown in Figure 5c and Figure 6A. Zeta-potential values were measured to study the stability of particles in aqueous medium, as shown in Figure 6B. The presence of MoS2 nanosheets shows good stability in an aqueous medium with a large negative zeta-potential value (ζ = −72.4 mV); even when the NIMO was precipitated with MoS2 nanosheets, the zeta potential shows negative value as showing in Figure 6B. The encapsulation of MoS_2_-NIMO composite using CHIT-SH results in a significant conversion of the zeta potential to a positive value, confirming the success of the encapsulation process. The encapsulation of NIMO-MoS_2_ composite by using the CHIT-SH shows converting in the zeta potential to a positive value, confirming the successful encapsulation process (means the modified chitosan was the shell for composite as core) and showing good stability as well in aqueous medium wherein the ζ = +70.7 mV for MoS_2_-NIMO-CHIT-SH composite. The stability of MoS_2_ nanosheets, MoS_2_-NIMO composite, and MoS_2_-NIMO-CHIT-SH composite was investigated using the sedimentation approach by using the turbidimeter measurement in water and PBS buffer solution as shown in Figure 6C and Figure 6D, respectively. The MoS_2_-NIMO-CHIT-SH composite shows higher stability compared to the other composites in both media with a decrease in relative turbidity with ~3.4% in water and ~11.1% in PBS buffer solution after 150 min.

Based on the results from Figure 5 and Figure 6, the encapsulation of NIMO with modified chitosan provides a good strategy to increase the stability and reduce the particle size of bare NIMO and can be used to control the drug release based on NIR properties of MoS_2_ as we see in the next section.

**Figure 7:**
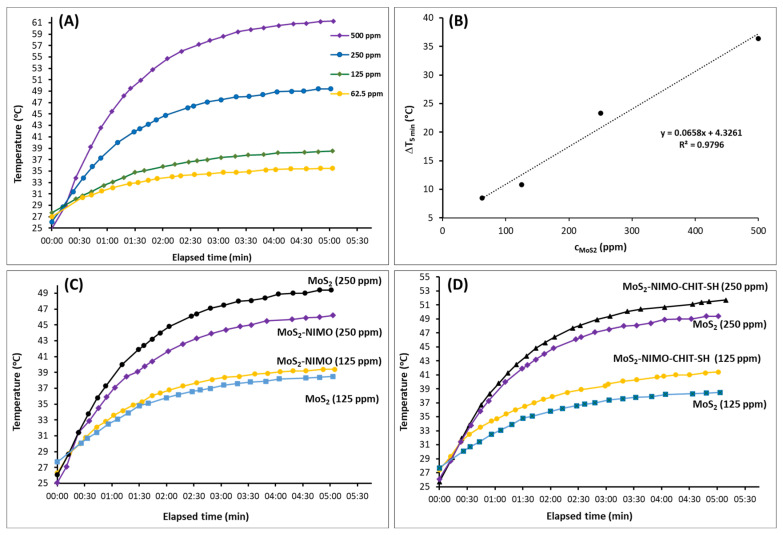
Photothermal excitation experiments: (**A**) temperature changes as a function of time at different MoS_2_ concentrations; (**B**) empirical relationship to the difference in temperature change (ΔT_5min_) as a function of MoS_2_ concentrations, the dash line displays the fitting line; (**C**) comparison of the temperature increase of MoS_2_ and MoS_2_-NIMO at different concentrations; and (**D**) between MoS_2_ and MoS_2_-NIMO-CHIT-SH composite at different concentrations.

Since the release efficiency and, thus, the expected therapeutic effect of the encapsulated NIMO drug depends on the photothermal conversion of composite NPs, the NIR light-induced temperature rise of the samples was studied. Figure 7A shows the temperature increase induced by the NIR laser light radiation as a function of elapsed time at different MoS_2_ concentrations. The temperature of MoS_2_ aqueous dispersion gradually increased from the 25–27 °C initial temperature, and they reached their maximum (plateau) values after about 2.5–3 min. The concentration dependence of the temperature rise is clearly visible, as shown in Figure 7B. The temperature rise values measured after 5 min of NIR light irradiation changed linearly with the MoS_2_ concentration. Figure 7A also shows that the initial slope of the curves, i.e., the kinetics of the temperature increase (°C/min), also changes with the concentration. In this way, the desired temperature rise can be easily controlled; thus, a temperature that is too high, which would result in damaging the organism, can be avoided. Figure 7C,D represents the rise in the NIR light-induced temperature NIMO and/or CHIT-SH containing samples. These samples also showed clear photothermal properties with concentration-dependent temperature increases. Moreover, the MoS_2_-NIMO-CHIT-SH composites produced a slightly higher temperature rise under the same conditions than the initial MoS_2_ (Figure 7D), which is presumably due to the higher degree of dispersity.

**Figure 8:**
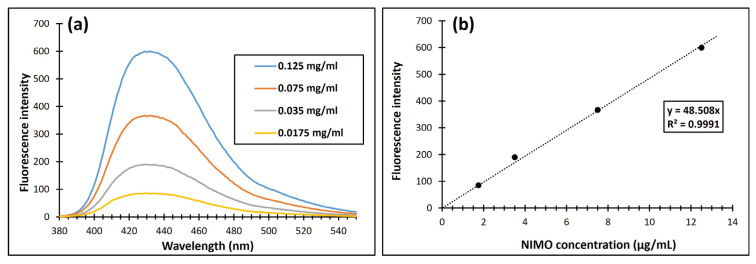
(**a**) Fluorescence intensity; and (**b**) calibration curve for NIMO by fluorescence measurements at λ_max_ = 431 nm, the dash line displays the fitting line.

Next, the study also examined how this NIR laser light-induced temperature increase manifests in the drug release properties. The calibration curve of NIMO was determined by a specific spectrofluorometric method. The method is based on the reduction of NIMO with Zn/HCl and measuring the obtained fluorescence at 431 nm after excitation at 360 nm. Using different concentrations range of stock solutions (0–125 µg/mL) of NIMO and measuring corresponding fluorescence intensity, the calibration curve can be determined as shown in Figure 8. Linear regression analysis of the data gave the following equation:

FI _431nm_ = 48.508 × C_NIMO_ (μg/mL); R^2^ = 0.9969

where FI is the fluorescence intensity, C is the concentration in mg/mL, and R is the correlation coefficient.

**Figure 9:**
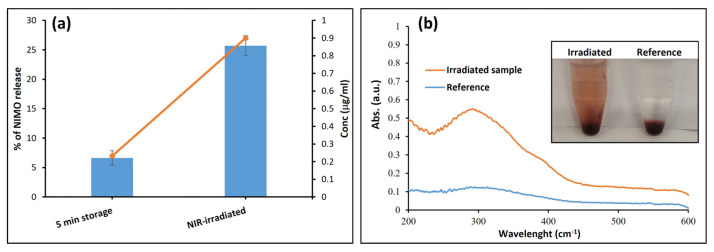
Photodynamic effect of MoS_2_ on active ingredient release; (**a**) in vitro release of NIMO drug from composite without NIR-radiation (as control) and with 5 min NIR-radiation (as a test), the orange line is to facilitate the visualization of the difference in the amount of drug released into the systems (**b**) UV-Vis absorption spectra of Sudan IV (a lipophilic azo dye) released from the composite storage (as control) and NIR illumination (as a test).

Based on this equation, the release of the NIMO drug can be determined in a simple, sensitive, and specific way. To investigate our idea that the release of NIMO can be triggered by using the NIR-irradiation approach, the concentration of the NIMO drug was determined in the presence and absence of NIR-irradiated, as shown in Figure 9a. The NIMO release in the NIR-irradiated sample was 25% (0.899 µg/mL) after 1 min irradiation, while the NIMO release (dissolution) in the non-irradiated samples was only 5% (0.23 µg/mL). To support and visualize our hypothesis, Sudan IV, as a poorly water-soluble lipophilic model dye, was added to the same system in place of the NIMO drug. By comparing the absorbance of the irradiated and non-irradiated samples, we found that the irradiation of MoS_2_ contributed to the diffusion of the poorly water-soluble dye and an increase in solution intensity, as seen in Figure 9b.

Since the case here may be a little different, as we are trying to prove that NIR light is the only control of drug release from the encapsulated system, we did not present the release curve as we typically see in drug delivery work. Instead, we presented this type of column to show the readers what is different when we apply NIR light and without it. Another issue is that the Spectro fluorescence technique of determining nimodipine takes a long time because we must perform the reduction reaction, quench it, and then measure the concentration of reduced nimodipine. Additionally, the NIMO can be decomposed by light, and applying numerous laser radiation cycles can cause the drug to decompose. For this reason, we offer our technology as a once-applied drug delivery system that can release a medicine at a concentration high enough to treat stroke.

**Figure 10:**
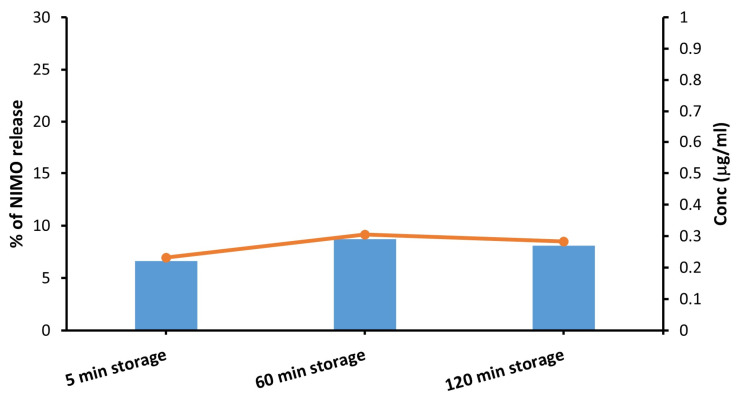
In vitro NIMO percentage release from the composite particles with different storage times (without NIR light irradiation), the orange line is to facilitate the visualization of the difference in the amount of drug released into the systems.

To further prove our idea that the NIR light irradiation of MoS_2_-containing composite particles is responsible for the release of the encapsulated drug, the NIMO concentration was compared at different storage times without NIR irradiation, as shown in Figure 10. As storage time increases, the increase in drug release becomes negligible, which means that the drug in its encapsulated form can be protected for a long time in an aqueous medium and can only be released by applying NIR radiation.

Loading NIMO and MoS_2_ nanosheets on the CHIT-SH can prolong the residence time of NIMO in the target site. MoS_2_-NIMO-CHIT-SH nanocomposites can respond to the NIR light in vitro and trigger drug release through photothermal conversion, enabling remote control of NIMO efficient release in the target site on demand by adjusting the radiation behavior of the NIR light. Injecting our composite into the target site and applying the NIR radiation allow for treatment-site-specific delivery of NIMO to treat stroke, migraine, Alzheimer’s disease, cerebrovascular spasm, and hypertension at lower systemic doses and significantly improve the treatment efficiency of NIMO. According to the results, MoS_2_-NIMO-CHIT-SH nanocomposites show strong NIR absorbance, which makes them a promising candidate for photothermal therapy. We present our approach as a once-applied drug delivery system capable of releasing medication at a level high enough to treat stroke. In future work, we plan to conduct a detailed study of the bio- and biological application of our presented system to see how feasible our system can be, as well as try to reduce the particle size of our system to increase the diverse application and cross the blood-brain barrier (BBB).

## 3. Materials and Methods

### 3.1. Materials

Low molecular weight (50–190 kDa) chitosan (CHIT, C_12_H_24_N_2_O_9_, ≥75% deacetylation) was purchased from Merck Ltd. (Darmstadt, Germany). 1-(3-Dimethylaminopropyl)-3-ethyl carbodiimide hydrochloride (EDC, C_8_H_17_N_3_·HCl, M_w_ 191.70, 98+%) was obtained from Thermo Fisher GmbH (Kandel, Germany), while 3-mercaptopropionic acid was purchased from Fluka Analytical (Munich, Germany). Nimodipine (NIMO, C_21_H_26_N_2_O_7_, ≥97.5%) and Molybdenum (IV) sulfide (MoS_2_, 98%) were purchased from Thermo Fisher GmbH (Kandel, Germany). Phosphate-buffered saline (PBS; pH~7.4) solution was prepared using sodium dihydrogen phosphate monohydrate (NaH_2_PO_4_·H_2_O) from Sigma-Aldrich (St. Louis, MO, USA), as well as di-sodium hydrogen phosphate dodecahydrate (Na_2_HPO_4_·12H_2_O) and sodium chloride (NaCl) acquired from Molar Chemicals Kft., Halásztelek, Hungary. Isopropanol (C_3_H_8_O, 99.9%) as an exfoliation solvent was purchased from Molar Chemicals Kft., Hungary. All chemicals were used exactly as supplied, with no additional purification.

### 3.2. Synthesis of 3-Mercaptopropionate Chitosan (CHIT-SH)

The thiolated chitosan (CHIT-SH) was synthesized by conjugation of 3-mercaptopropionic acid with chitosan according to our previous work [43]. Briefly, 25 mL 2% *w*/*v* of low M_w_ chitosan in acetic acid was mixed with 1.2 mL of 3-mercaptopropionic acid (13.7 mmol), then 2.68 g of EDC (13.7 mmol) was added to the reaction mixture, and the reaction was magnetically stirred for 6 h at room temperature under the dark. After the reaction time, the reaction mixture was added to an excess amount of ethanol (~200 mL), then the product was collected by centrifugation (5000 rpm × 30 min), and then washed with ethanol several times, and eventually dried under vacuum.

### 3.3. Preparation of MoS_2_ Nanosheets

MoS_2_ nanosheets were obtained by using the liquid phase exfoliation technique. First, 100 mg of MoS_2_ powder was dispersed in 40 mL of isopropanol and sonicated at 37 °C in a constant temperature water bath for 5 h. To separate MoS_2_ nanosheets and powders, the supernatant containing MoS_2_ nanosheets was obtained by a low-speed (1500 rpm × 10 min) centrifugation, and the solvent was removed by high-speed (5000 rpm × 15 min) centrifugation, and then the collected product was dried under vacuum.

### 3.4. Preparation of MoS_2_-NIMO and MoS_2_-NIMO-CHIT-SH Composites

For the preparation of the MoS_2_-NIMO composite, 1.2 mg of MoS_2_ nanosheets were dispersed in 2.5 mL of ethanol by sonication and stirring, followed by dropwise adding 41.8 μg of NIMO drug (1 mg/mL in ethanol) for ethanolic dispersion of MoS_2_ nanosheets under continuously magnetic stirring. In the next step, 10 mL of H_2_O was dropwise added to the dispersion to precipitate the composite under strong stirring. The product was collected by Lyophilization. The mass percentage composition of the composite was also determined: 96.63% MoS_2_ and 3.37% NIMO. In the case of the MoS_2_-NIMO-CHIT-SH composite, the preparation was carried out according to the aforementioned process. Here, the difference was the addition of 10 mL of aqueous 0.5% *w*/*v* CHIT-SH instead of 10 mL of H_2_O to the MoS_2_-NIMO ethanolic dispersion under strong magnetic stirring. Mass percentage composition for the three-component composite: 2.34% MoS_2_, 0.08% NIMO and 97.58% CHIT-SH.

### 3.5. Methods of Characterization

Fourier Transform Infrared (FTIR) spectroscopy measurement was used to examine the initial chitosan and modified form (CHIT-SH). The spectra were obtained using an AVATAR330 FTIR spectrometer (Thermo Nicolet, Unicam Hungary Ltd., Budapest, Hungary) integrated with a deuterated triglycine sulfate detector and set at 400–4000 cm^−1^. The spectral resolution was adjusted to 2 cm^−1^, and 128 scans were carried out to improve the signal-to-noise ratio. The background was recorded with a pure KBr disk and subtracted from each spectrum.

The ^1^H-NMR spectra were recorded in D_2_O as the solvent in 5 mm tubes at room temperature (RT) on a Bruker DRX-500 spectrometer (Bruker Biospin, Karlsruhe, Baden Württemberg, Germany) at 500 (1H) MHz, with the deuterium signal of the solvent as the lock and TMS as internal standard (1H).

A transmission electron microscope (TEM) was utilized to examine the particle size and the morphology of the bare NIMO and the composites. TEM analysis was performed using a FEI TECNAI G^2^ 20 X-Twin high-resolution transmission electron microscope operating at an acceleration voltage of 200 kV. 10 µL of the samples were pipetted onto a 200 mesh copper grid with carbon-coated film (CF200-Cu, Electron Microscopy Sciences, Hatfield, PA, USA). The samples were dried at room temperature.

### 3.6. Study the Precipitation Properties of NIMO and Its Composites

The precipitation of the NIMO drug and its composite forms was carried out to determine the precipitation point and figure out the scheme for the preparation of our final composite. For this, 2.5 mL of 0.1% *w*/*v* NIMO was prepared in ethanol, followed by the dropwise addition of 10 mL of distilled water under strong magnetic stirring and monitoring the change in the absorbance due to the formation of the precipitated particles by using an Ocean Optics USB2000 UV-VIS Spectrometer (Ocean Optics Ins., Seminole, FL, USA) with a 1 cm quartz cuvette at λ = 550 nm. In the case of the NIMO-MoS_2_ composite, 2.5 mL of ethanol-based NIMO (c = 0.1%) and MoS_2_ (c = 0.00145%) was precipitated by adding 10 mL of distilled water, and the precipitation was followed by absorbance measurements. In the case of the NIMO-MoS_2_-CHIT-SH composite, 2.5 mL of ethanol-based NIMO (c = 0.1%) and MoS_2_ (c = 0.00145%) was precipitated by adding 10 mL of 0.5% aqueous CHIT-SH solution under vigorous magnetic stirring at 25 °C and the absorbance was continually measured and recorded as a function of the added volume of solution.

### 3.7. Study the Stability of MoS_2_, MoS_2_-NIMO and MoS_2_-NIMO-CHIT-SH in the Aqueous Medium

By using sedimentation measurement, the stability of MoS_2_ nanosheet, MoS_2_-NIMO, and MoS_2_-NIMO-CHIT-SH particles in an aqueous medium was assessed as follows: 10 mL of distilled water and phosphate buffer saline solution (PBS) were used to prepare 0.03% *w*/*v* of the sample. The resulting dispersion was sonicated for 5 min and then promptly placed in a turbidimeter. An ISO-HI98703 portable turbidimeter (Hanna Instruments, Cluj, Romania) was used to track the sedimentation of the dispersion at 25 °C. Without stirring, the turbidity was carried out in a steady-state situation. The measurement was conducted three times, and the standard deviation was calculated and presented as error bars.

The hydrodynamic radius and zeta potential values of the MoS_2_ nanosheet, MoS_2_-NIMO, and MoS_2_-NIMO-CHIT-SH particles dispersion were measured using dynamic light scattering (DLS) using Nano Partica SZ-100 (HORIBA Scientific, Kyoto, Japan) in sterilized water and PBS buffer solution (pH 7.4). Five repetitions of the measurements were conducted at 25 ± 0.1 °C in order to calculate the standard deviation.

### 3.8. Characterization and Evaluation of Photothermal Property

A Fluke-type thermal camera (Fluke Ti400 Pro, Everett, WA, USA) was applied to take the photothermal image of MoS_2_-containing samples at different times under NIR radiation. A NIR light laser source (RLT915-1000GOP) with a wavelength of 915 nm and constant (10 W/cm^2^) power density was used to irradiate a sample cell (Eppendorf tube) containing 20 mL of sample dispersion with different concentrations (62.5, 125, 250, and 500 ppm, respectively). The temperature change of the system was measured every 30 s for 5 min.

### 3.9. In Vitro Drug Release Experiments

To determine the NIMO ingredient content, it was first necessary to prepare a calibration solution series. A stock solution of NIMO was prepared by dispersing 1 mg of the active ingredient in 0.5 mL of ethanol and then adding 10 mL of H_2_O. The dissolution in ethanol was necessary because NIMO is poorly soluble in water. Next, the members of the solution series were prepared by diluting with an appropriate volume of water to have 1 mL, with concentrations of 1.75, 3.50, 7.50, and 12.5 μg/mL. In each member of the solution series, a chemical transformation was required regarding the structure of NIMO. Since the nitro group (–NO_2_) of NIMO does not provide fluorescent activity, it had to be reduced to an amino group (–NH_2_). The addition of 0.1 g Zn and 2 mL of HCl (c = 1 M) resulted in the evolution of hydrogen, which completed the reduction of NIMO in the solution series. After 15 min of standing in a dark environment, the systems were treated with 3 mL of Triton X-100 (2% *v*/*v* aqueous solution) surfactant solution to quench the reaction. The system was diluted to 10 mL by distilled water, followed by filtration using a cellulose membrane (Munktell & Filtrak GmbH, Bärenstein, Germany; grade 390), and their fluorescent activity was measured at 360/431 nm [59]. The calibration curve was recorded by spectrofluorimetric measurement, wherein the linear regression analysis of the data gave the following equation:

C_NIMO_ (μg/mL) = FI _431nm_/48.508; R^2^ = 0.9969

where FI is the fluorescence intensity, C is the concentration in mg/mL, and R is the correlation coefficient.

To determine the release of drug from the NIMO-loaded photodynamic composite, 0.5425 mg of composite in 100 µL of ethanol/water (0.5 mL + 10 mL), followed by a reduction reaction by using 0.05 g of Zn and 1 mL of 1N HCl for 15 min, then 1.5 mL of 2% Triton solution added, and the system was diluted to 5 mL by distilled water. The mixture was filtrated, and the relative fluorescence of the filtrate was measured at 360/431 nm. The same measurement was repeated in the case of the irradiated sample, and different storage times were used for the non-irradiated sample as well.

## 4. Conclusions

In the current work, a novel drug delivery nanosystem consisting of MoS_2_-NIMO-CHIT-SH was successfully constructed to facilitate the NIR light-induced release of NIMO drugs. The successful modification of initial chitosan (CHIT) to produce thiolated chitosan (CHIT-SH) was confirmed by FTIR and ^1^H-NMR measurements. The prepared nanosystem showed good stability in the aqueous medium, as confirmed by turbidity measurements for 150 min and zeta potential measurements. Encapsulation of NIMO into CHIT-SH resulted in the formation of well-defined spherical nanoparticles of NIMO-loaded photodynamic composite with an average particle size of around 500 nm. The drug delivery nanosystem responded to NIR light and triggered drug release through photothermal conversion, which enabled the remote control of the highly efficient release of NIMO by adjusting the radiation behavior of the NIR light. The NIR radiation at 1 W/cm^2^ could increase the drug release efficiency of NIMO to 25% (0.899 µg/mL) compared to 5% for non-irradiated samples. NIR radiation was the only controlled system for drug release, as proven by the storage time experiment.

## Data Availability

The data presented in this study are available in the article.

## Abbreviations

The following abbreviations are used in this manuscript:

CHIT	Chitosan
CHIT-SH	Thiolated chitosan
NIR	Near-infrared
NIMO	Nimodipine
EDC	1-(3-Dimethylaminopropyl)-3-ethyl carbodiimide hydrochloride
PBS	Phosphate buffer saline solution
DS	Degree of substitution
DD	Degree of deacetylation
FTIR	Fourier Transform Infrared
TEM	Transmission electron microscope
DLS	Dynamic light scattering
